# High Expression of long non-coding RNA PVT1 predicts metastasis in Han and Uygur Patients with Gastric Cancer in Xinjiang, China

**DOI:** 10.1038/s41598-018-36985-x

**Published:** 2019-01-24

**Authors:** Xianxian Ren, Dongdong Cao, Li Yang, Xia Li, Wei Zhang, Yongbiao Xiao, Yu Xi, Feng Li, Dongmei Li, Zemin Pan

**Affiliations:** 1 School of Medicine, Shihezi University, Shihezi, Xinjiang China; 2 First Affiliated Hospital of Shihezi University, Shihezi, Xinjiang China; 3 First People’s Hospital of Kashi, Kashi, Xinjiang China; 4 Department of Pathology, Beijing Chaoyang Hospital, Capital Medical University, Beijing, China

## Abstract

To analyze the level and diagnostic value of plasmacytoma variant translocation 1 (PVT1) in gastric cancer (GC) of Han and Uygur in Xinjiang, China, we collected 42 GC and 47 normal gastric tissues and performed tissue microarray. *In situ* hybridization was used to detect PVT1, while immunohistochemistry was used to analyze c-myc. The relationship between PVT1, c-myc and clinical pathological features was investigated. We then analyzed the expression of PVT1 in six GC cell lines. RNA interference was used to silence PVT1 in BGC823 and AGS cells. c-myc was detected by western blotting after silencing PVT1, while proliferation, invasion and migration ability were also analyzed. We found that PVT1 and c-myc were highly expressed in both Han and Uygur GC tissues. In Han GC, PVT1 was correlated with lymph node metastasis and primary tumor site. In Uygur GC, both PVT1 and c-myc were correlated with lymph node metastasis and clinical staging. PVT1 was positively correlated with c-myc. BGC823 and AGS cells exhibited high levels of PVT1. When PVT1 expression was silenced, the expression of c-myc decreased, while migration and invasion ability were also decreased in cells. PVT1 could therefore be a potential biomarker to predict the metastatic tendency of GC in both Han and Uygur patients.

## Introduction

Gastric cancer (GC) is the second most common cause of cancer death worldwide and GC mortality in China accounts for 42% of all GC deaths worldwide. The incidence and mortality rates of GC in 2006 were 35.02 and 26.08 per 100,000 persons, respectively, in China^1^. Cancer statistics in 2015 showed 679,100 new GC cases and 498,000 deaths. The incidence and mortality of GC in China is currently second only to lung cancer with the highest burden found in southwest China^2^.

Early symptoms of GC are not obvious. In most GC patients, the disease has progressed to the midor advanced stage when they are diagnosed. However, the prognosis of GC is closely associated with the TNM stage. The 5-year survival rates of GC patients are 90%, 50–60%, and 10–15% in GC Stages I, II and III, respectively^3^; thus it is important to identify diagnostic and predictive markers of GC. This would help patients to select a suitable treatment method and carry out correlative checks to avoid the full extent of the risks. Therefore, tumor biomarkers would be a significant help in prolonging the life of GC patients. Several potential approaches have been used to identify suitable biomarker candidates^4^; however, their sensitivity and specificity are limited.

Long non-coding RNAs (lncRNAs) are greater than 200 nucleotides in length. They play roles in epigenetic gene regulation, transcriptional regulation and the gene expression process at transcription and post-transcription levels^5^. Thus they have several significant functions in the fundamental biological processes of cells and are emerging as new players in the tumorigenic process^6^. LncRNAs exhibit specific expression in tissues and can be detected easily. This property makes them ideal candidates for biomarkers^7^. Consequently lncRNAs such as *H19*^8,9^, *GAS5*^10^, and *HOTAIRM1*^11,12^ have received significant attention for the early diagnosis of cancer. Plasmacytoma variant translocation 1 (PVT1) is a new lncRNA, located on chromosome 8q24 upstream of the oncogene MYC. It has been reported that PVT1 maybe related to GC and could be a potential lncRNA biomarker for GC^13,14^.

Xinjiang is the largest autonomous region in northwestern China. The population in this region is composed of a number of ethnic groups. The major ethnic groups are the Han (39.7%) and the Uygur (45.7%). The incidence rate of GC in Uygur is 12.76% (351/2751) and in Han people is 3.85% (92/2568)^15,16^. In Han and Uygur GC patients, the histopathologic features are the same, but whether the diagnostic parameters, especially some biomarkers at the genetic and molecular biology level, are suitable for both ethnic groups remain unknown. It is also not known whether the PVT1 expression level shows the same tendency in Han and Uygur GC patients.

In this study, we investigated the expression level of PVT1 and c-myc in GC tissues of Han and Uygur patients by *in situ* hybridization (ISH) and immunohistochemistry (IHC). We explored the relationship between the expression of PVT1 and c-myc and clinicopathological features. We further analyzed the effects of PVT1 on the proliferation, migration, and invasive ability of GC cells and analyzed the clinical value of PVT1 as a biomarker for the diagnosis and prognosis of Han and Uygur GC patients.

## Results

### PVT1 is over expressed in GC tissues and is closely related to lymph node metastasis in both Han and Uygur GC patients

**Figure 1:**
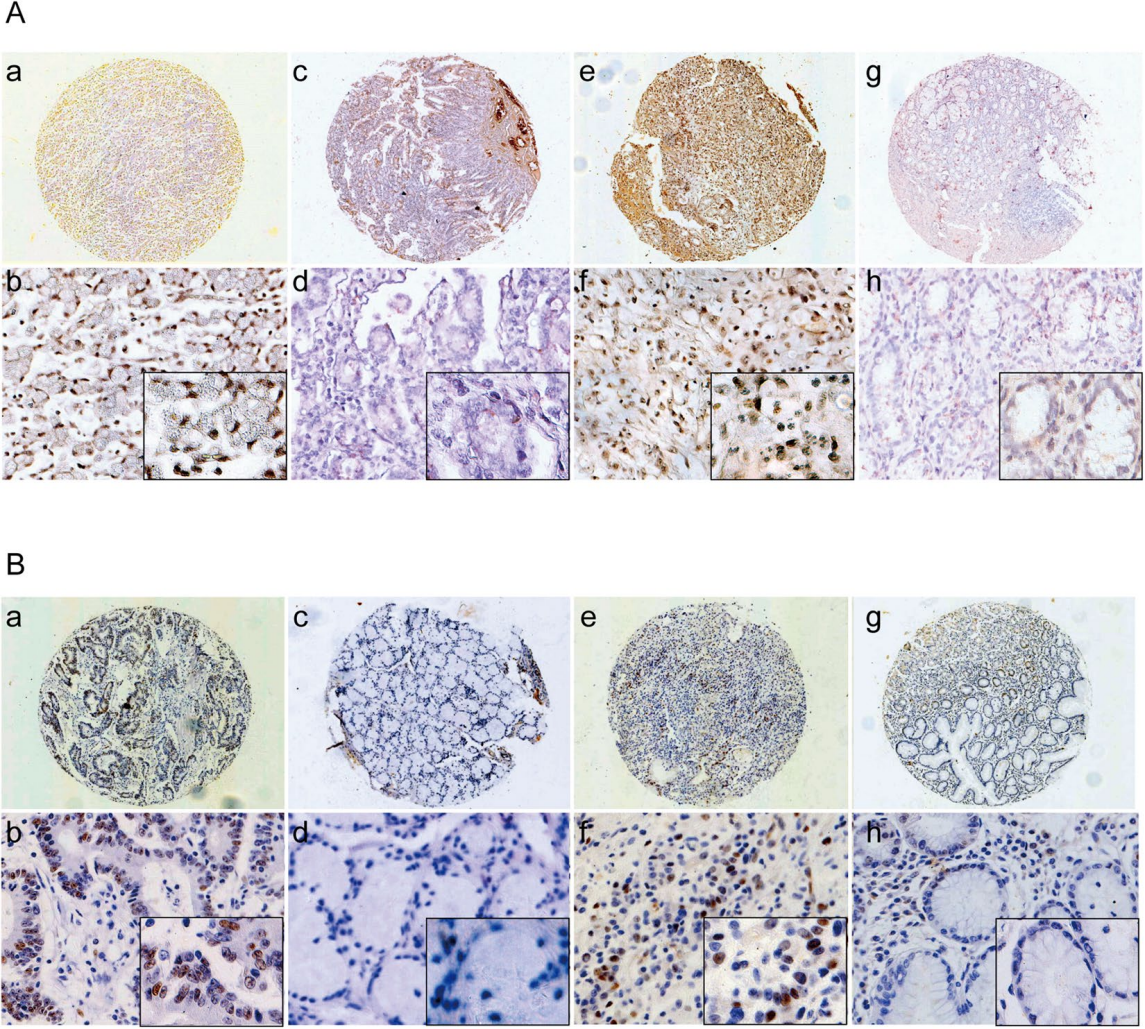
Comparison of the expression of lncRNA PVT1 and c-myc in GC and normal tissues by ISH and IHC. (**A**) Comparison of the expression of lncRNA PVT1 in GC and normal tissues by TMA and ISH. PVT1 staining was stronger in GC tissues. (a) PVT1 staining in Han GC tissues (40×); (b) PVT1 staining in Han GC tissues (200×, 400× in the lower right corner); (c) PVT1 staining in Han normal gastric tissues (40×); (d) PVT1 staining in Han normal gastric tissues (200×, 400× in the lower right corner); (e) PVT1 staining in Uygur GC tissues (40×); (f) PVT1 staining in Uygur GC tissues (200×, 400× in the lower right corner); (g) PVT1 staining in Uygur normal gastric tissues (40×); (h) PVT1 staining in Uygur normal gastric tissues (200×, 400× in the lower right corner). (**B**) Comparison of c-myc expression in GC and normal tissues by TMA and IHC. Staining of c-myc was stronger in GC tissues. (a) c-myc staining in Han GC tissues (40×); (b) c-myc staining in Han GC tissues (200×, 400× in the lower right corner); (c) c-myc staining in Han normal gastric tissues (40×; (d) c-myc staining in Han normal gastric tissues (200×, 400× in the lower right corner); (e) c-myc staining in Uygur GC tissues (40×); (f) c-myc staining in Uygur GC tissues (200×, 400× in the lower right corner); (g) c-myc staining in Uygur normal gastric tissues (40×); (h) c-myc staining in Uygur normal gastric tissues (200×, 400× in the lower right corner).

After ISH, taking into account samples which were lost from the slides, the remaining samples comprised 14 Han GC tissues, 15 Han normal gastric tissues, 15 Uygur GC tissues, and 16 Uygur normal gastric tissues, all of which were analyzed for PVT1 expression. The results showed that PVT1 was located in both cytoplasm and nucleus (Fig. 1A). The PVT1 expression level in normal gastric tissues was lower than that of GC tissues in both Han and Uygur patients (*P* < 0.005; Fig. 1A). The positive expression rate of PVT1 in Han GC was 71.42% (10/14), and 26.67% (4/15) in normal gastric tissues, a difference which was statistically significant (*P* < 0.05; Fig. 1A, Table 1). Meanwhile the positive expression rate of PVT1 in Uygur GC tissues was 53.33% (8/15), and in gastric tissues, it was 12.50% (2/16), which was also statistically significant (*P* < 0.05; Fig. 1A, Table 1). There were no significant differences in the expression of PVT1 between Han and Uygur patients (*P* = 0.268). Serum from four different ethnic populations showed that PVT1’s levels were highest in Uygur people (*P* = 0.0211). PVT1 serum levels in Uygur and Hui people were statistically significantly different (*P* = 0.0044) (Supplement Fig. 1).

**Table 1:** The expression of PVT1 in normal gastric mucosa and gastric cancer (GC) tissues in Han and Uygur.

Group	n	Han		Positive rate	*P*	n	Uygur		Positive rate	*P*
		negative	positive				negative	positive		
Normal	15	11	4	26.67%	0.027	16	14	2	12.50%	0.023
GC	14	4	10	71.42%		15	7	8	53.33%	

For the analysis of clinicopathological characteristics, in Han GC patients, PVT1 expression levels correlated with lymph node metastasis and primary tumor site (*P* < 0.05). In Uygur GC patients, PVT1 expression levels were related to lymph node metastasis and clinical staging (*P* < 0.05; Table 2). No significant correlation was found between PVT1 expression and gender, age at diagnosis, or histopathological grade (Table 2).

**Table 2:** The relationship between the expression of PVT1 and clinicopathological features of GC patients.

Group		n	Han		*P*	n	Uygur		*P*
			negative	positive			negative	positive	
Sex	male	7	3	4	0.559	10	5	5	1.000
	female	7	1	6		5	2	3	
Age (years)	≤55	6	1	5	0.580	4	3	1	0.282
	➢55	8	3	5		11	4	7	
T	T_1−2_	3	3	0	0.033	2	1	1	1.000
	T_3–4_	11	1	10		13	6	7	
N	N_0_	4	3	1	0.041	6	5	1	0.041
	N_1−3_	10	1	9		9	2	7	
Stage	I II	3	2	1	0.176	6	5	1	0.041
	III IV	11	2	9		9	2	7	
Histopathological grade	Well + Moderately differentiated	6	2	4	1.000	6	1	5	0.119
	Poorly differentiated	8	2	6		9	6	3	

Note: aFisher’s exact probability test. T: the primary tumor site; N: the involvement of regional lymph nodes; M: the presence of distant metastases; *P < 0.05.

### C-myc is over expressed in both Han and Uygur GC tissues

The expression levels of c-myc were analyzed in 22 Han GC tissues, 23 Han normal gastric tissues, 20 Uygur GC tissues, and 24 Uygur normal gastric tissues using IHC. The results showed that normal gastric tissues have a lower c-myc expression levels and GC tissues had higher levels in both Han and Uygur individuals (*P* < 0.05; Fig. 1B, Table 3). The positive expression rate of c-myc in the Han GC tissues was 59.09% (13/22), while it was 17.39% (4/23) in normal gastric mucosa (*P* < 0.05; Fig. 1B, Table 3). The positive expression rate of c-myc in Uygur GC tissues was 65% (13/20), and in normal gastric tissues it was 29.17% (7/24) (*P* < 0.05; Fig. 1B, Table 3). There were no significant differences in c-myc expression between Han and Uygur populations (*P* = 0.471).

**Table 3:** The expression of c-myc in normal gastric mucosa and GC in Han and Uygur.

Group	n	Han		Positive rate	P	n	Uygur		Positive rate	P
		negative	positive				negative	positive		
Normal	23	19	4	17.39%	0.006	24	17	7	29.17%	0.032
GC	22	9	13	59.09%		20	7	13	65%	

Analysis of clinicopathological characteristics revealed that c-myc expression levels correlated with histopathological grade in Han GC patients, while these levels were correlated with lymph node metastasis and clinical staging in Uygur GC patients (*P* < 0.05; Table 4). No correlation was found between c-myc expression and gender or age (Table 4).

**Table 4:** The relationship between the expression of c-myc protein and clinicopathological features of patients with GC.

		n	Han		*P*	n	Uygur		*P*
			negative	positive			negative	positive	
Sex	male	15	6	9	1.000	13	6	7	0.329
	female	7	3	4		7	1	6	
Age (years)	≤55	7	3	4	1.000	8	1	7	0.158
	>55	15	6	9		12	6	6	
T	T_1–2_	6	3	3	0.655	2	2	0	0.111
	T_3–4_	16	6	10		18	5	13	
N	N_0_	7	3	4	1.000	7	5	2	0.022
	N_1–3_	15	6	9		13	2	11	
Stage	I II	8	5	3	0.187	5	4	1	0.031
	III IV	14	4	10		15	3	12	
Histopathological grade	Well + Moderately differentiated	10	7	3	0.027	6	2	4	1.000
	Poorly differentiated	12	2	10		14	5	9	

Note: aFisher’s exact probability test. T: the primary tumor site; N: the involvement of regional lymph nodes; M: the presence of distant metastases; *P < 0.05.

### PVT1 levels are related to c-myc expression

The relationship between the expression levels of PVT1 and c-myc were analyzed to identify possible mechanisms. We stratified the results of PVT1-positive cases and analyzed the c-myc expression levels in the corresponding site to see if there was any association between PVT1 and c-myc. The results showed that PVT1 expression was associated with c-myc (r = 0.546, *P* = 0.005, Table 5).

**Table 5:** Correlation analysis between PVT1 and c-myc.

	c-myc^−^	c-myc^+^	Correlation coefficient	*P* value
PVT1^−^	7	2	0.546	0.005
PVT1^+^	2	14		

### PVT1 expression levels are higher in BGC823 and AGS GC cells

**Figure 2:**
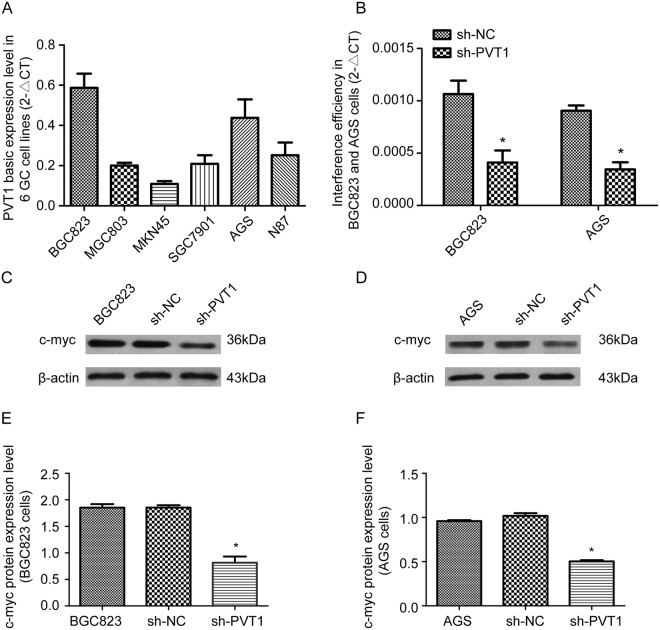
Decreased c-myc expression in BGC823 and AGS cells after interference with PVT1 expression. (**A**) Real time-PCR results show the endogenous PVT1 expression levels in the six GC cell lines BGC823, MGC803, MKN45, SGC7901, AGS and N87. (**B**) RNAi was used to interfere with the expression of PVT1 in BGC823 and AGS cells, and then the efficiencies of PVT1 knockdown were investigated using real time-PCR. (**C**,**D**) Detection of c-myc protein expression levels by western blotting in BGC823 and AGS cells after silencing of PVT1. *P < 0.05.

To select a suitable cell line for further research, the basic expression level of PVT1 was analyzed in six GC cell lines: AGS, SGC7901, N87, MGC803, BGC823 and MKN45, and expression was found to differ between the cell types (Fig. 2A). Real-time PCR revealed higher levels of endogenous PVT1 in BGC823 and AGS cells. These two cell lines were therefore selected for subsequent experiments (Fig. 2A). Four PVT1 short hairpin RNA (shRNA) plasmids and a control plasmid were used to transfect BGC823 and AGS cells and the efficiencies of interference were analyzed with real time-PCR (Fig. 2B). The results showed that sh-PVT1-4 effectively down-regulated expression of PVT1. Consequently, we selected sh-PVT1-4 for subsequent studies.

### PVT1 regulates c-myc expression in GC cells

To determine whether the expression levels of the lncRNA PVT1 caninfluence the expression of c-myc in GC cells, c-myc protein was analyzed with western blotting in the PVT1 down-regulated BGC823 and AGS cells (Fig. 2C–F). The results showed that, compared with normal BGC823 and AGS cells, c-myc protein expression was reduced in cells with knockdown of PVT1. This indicates that the lncRNA PVT1 contributes to the regulation of c-myc expression in GC cell lines (Fig. 2C–F).

### PVT1 down-regulation inhibits the invasion and migration ability of GC cells

**Figure 3:**
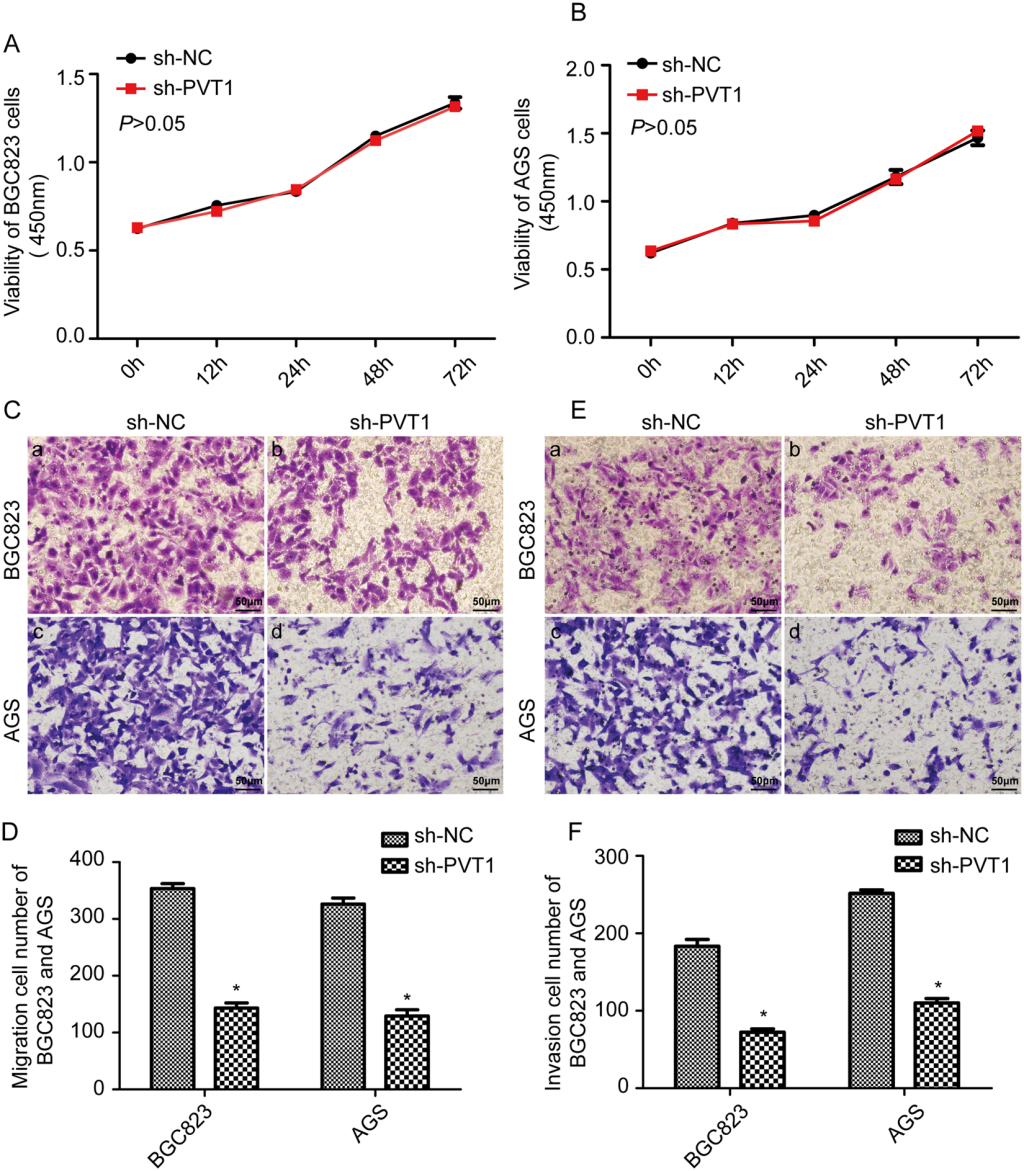
Down-regulation of PVT1 inhibited the invasion and migration ability of GC cells. (**A**,**B**) The results of CCK8 assay showed that knock down of PVT1 had no obvious effect on cell proliferation in BGC823 and AGS cells (*P* > 0.05). (**C**,**D**) Cell migration assays showed that interference with the expression of PVT1 suppressed the cell migration ability of BGC823 and AGS cells, *P < 0.05. (**E**,**F**) Matrigel invasion assays showed that interference with the expression of PVT1 suppressed the cell invasion ability of BGC823 and AGS cells. *P < 0.05.

To explore the potential effects of the lncRNA PVT1 on the pathogenesis of GC, the proliferative ability of PVT1 down-regulated BGC823 and AGS cells was analyzed with the Cell Counting Kit-8 (CCK8) assay. The invasion and migration abilities were analyzed by Transwell assay with or without Matrigel. The results of the CCK8 assay showed that interference with the expression of PVT1 in BGC823 and AGS cells does not obviously affect their proliferation ability (Fig. 3A,B, *P* > 0.05). Cell migration assays showed that down-regulation of the lncRNA PVT1 inhibited the migration ability of BGC823 and AGS cells compared with the sh-NC group (Fig. 3, *P* < 0.05). Furthermore, the Matrigel invasion assay showed that down regulated lncRNA PVT1 inhibited cell invasion compared with the sh-NC group (Fig. 3, *P* < 0.05).

## Discussion

GC is a major cause of cancer-related mortality in China^17^. The patient’s genetic background, bacterial virulence, environmental and many other factors have been implicated in affecting the gastric oncogenic process, but the underlying molecular mechanism remains poorly understood^18^. Many patients are diagnosed with advanced GC and the prognosis is poor. Screening for a sensitive biomarker for the diagnosis and prognostic estimation of GC would help to prolong survival^19^. Han and Uygur people in Xinjiang, China, have different genetic backgrounds, and their characteristics of morbidity and mobility in GC are also different^20^. Whether these biomarkers are as effective for diagnosis or prognosis estimation in the different ethnic groups is unknown.

In recent years, the results of transcriptomics studies have indicated that only approximately 2% of the genes comprising the human genome are protein-coding genes. The remaining 98% are transcribed into non-coding RNAs (ncRNAs). Among ncRNAs, 80% are lncRNAs^21^. Many studies have shown that lncRNAs are frequently deregulated in various cancers and that they exert multiple functions in cell proliferation, migration and invasion. Interestingly, lncRNAs could also be important biomarkers for clinical diagnosis and drug targeting in cancer^22^.

PVT1 is a 1957 bp linear lncRNA^23^ which consists of nine exons. Abnormal PVT1 expression has been shown to be a powerful predictor of cancer progression and patient survival in colorectal cancer, hepatocellular carcinoma and lung cancer^24^. Research has also shown that PVT1 is linked to GC. Cao *et al*.^13^ analyzed GC tissues using a microarray and found that the lncRNA PVT1 was up-regulated in tumor tissues^25^. Kong *et al*.^26^ further confirmed that PVT1 was up-regulated in GC tissues using real time-PCR. The results suggested that PVT1 silencing may be able to block the G1 phase of the cell cycle. Knockdown of PVT1 was reported to cause G1 phase arrest in clear cell renal cell carcinoma^27^ and melanoma cells^28^. PVT1 silencing decreases cyclin D1 expression. Also, PVT1 maybe associated with enhancer of zeste homolog 2, a histone methyltransferase and mediate epigenetic regulation. Similarly, PVT1 can repress members of the INK family p15 and p16. Zhang *et al*.^29^ found that PVT1 promotes multidrug resistance in GC cells. These results indicate that PVT1 is closely related to the mechanism and treatment of GC. However, its precise functions and underlying molecular mechanisms remain to be determined. We are also eager to assess whether PVT1 expression plays a significant role in the carcinogenesis of GC in patients of different races.

To answer these questions, in the present study, we collected tissues from Han and Uygur GC patients and normal persons. We aimed to investigate whether PVT1 is over-expressed in GC tissues from the two different ethnic groups. We also wanted to compare whether there are any significant differences between the cancer tissues of these two groups. Our results confirmed that PVT1 is up-regulated in GC tissues compared with normal gastric tissues in both Han and Uygur patients based on ISH, but there is no significant difference between the two groups. The PVT1 serum levels in four different ethnic groups were higher in Uygur than in the other groups. Additionally, we analyzed the relationship between expression of PVT1 and the clinical pathological characteristics, and the results suggest that PVT1 could be a marker for the diagnosis and assessment of the tendency for lymphatic metastasis of GC patients.

Metastasis and invasion are important factors and are responsible for most cancer-associated deaths. The 5-year survival rate of metastatic or advanced GC is only 5–15%. The median overall survival for these patients is less than 1 year. Many studies have elucidated the pathogenesis of primary tumor formation, but the metastatic mechanisms remain poorly understood^30^. Recently, there have been increasing reports demonstrating that lncRNAs are emerging as critical biomolecules for tumor metastasis. Meng *et al*.^31^ found that the lncRNA CRNDE promoted the cell growth and stimulated the metastasis of cervical cancer cells. Gao *et al*.^32^ investigated the functional role of GAS5 in pancreatic cancer metastasis and found that GAS5 positively regulated the PTEN-induced tumor-suppressor pathway. Jiang *et al*.^33^ indicated that HOTAIR was related to the metastasis of non-small cell lung cancer via regulation of miR-613 expression. However, no biomarkers for early detection and prediction of lymph node metastasis have yet been well established for GC^34^. Our results from Han and Uygur GC tissues indicated that PVT1 correlates with lymphatic metastasis. In a further assay at the GC cell level, we also demonstrated that interference with the expression of PVT1 increased cell metastasis and invasion ability. There have also been some other reports suggesting that PVT1 is related to metastasis in some cancers. Lan *et al*.^35^ found that PVT1 serves as a competing endogenous RNA for miR-186-5p, promoting the tumorigenesis and metastasis of hepatocellular carcinoma. Zhou *et al*.^36^ reported that PVT1 is involved in the epithelial-to-mesenchymal transition (EMT) of digestive system cancer. All of these observations mean that PVT1 is closely related to the metastasis and invasion ability of GC cells. GC patients who have a high PVT1 level in their tissues need to be vigilant regarding lymphatic metastasis.

In addition, lncRNAs can regulate the transcription of adjacent genes by combining with transcription factors or polymerases. PVT1 is located in the cancer-related region, chromosome 8q24, upstream of the oncogene MYC. PVT1 function may thus be closely related to c-myc. Our IHC analysis also showed that PVT1 expression is related to c-myc and showed that they have the same expression tendency. In PVT1-silenced GC cells, c-myc was also down regulated. Dysregulation of c-myc is a common feature in cancer, and recent studies have shown that c-myc has important roles in GC development and progression^37^. High expression of c-myc is correlated with advanced disease stage, lymph-node metastasis and poor survival rates^38^. Knockdown of c-myc inhibited the invasion and metastasis of GC cell lines *in vitro*^39^. MYC was also found to be an indirect regulator of metastasis in a c-RAF-driven mouse model of non-small cell lung cancer^40^. Another study found that functional inactivation of MYC in human breast cancer cells specifically inhibited distant metastasis *in vivo* and invasive behavior of these cells *in vitro*^41^. MYC sometimes helps other molecular pathways to promote cancer metastasis. Notch1 was reported to regulate invasion and metastasis of head and neck squamous cell carcinoma by inducing EMT through c-myc^42^. Yang *et al*. also found that PVT1 knockdown down regulated the expression of c-myc in prostate cancer cell lines^43^. PVT1 may act through up-regulation of the expression of c-myc to promote lymph-node metastasis and progression of GC.

In this study, c-myc was correlated with lymph node metastasis only in Uygur GC patients and not in Han GC patients. This may have been due to the different genetic backgrounds in different ethnic groups. The molecular mechanism of lymph node involvement and metastasis maybe different in these two ethnic groups. PVT1 was reported to regulate some molecules to promote involvement and migration. PVT1 can promote EMT and migration by down regulating p21 in pancreatic cancer cells^44^. EMT in cancer cells leads to the acquisition of invasive and metastatic properties. In prostate cancer, PVT1 can act as a sponge for miRNA-186-5p and positively regulates Twist1 to promote EMT^45^. PVT1 also decreases miR-195 expression by enhancing histone H3K27me3 and by direct sponging of miR-195 to modulate EMT in cervical cancer cells^46^. In gastric cancer cells, whether PVT1 is related to these molecules and its functions are undefined. PVT1 also encodes a wide variety of non-coding RNAs, including a cluster of six annotated microRNAs: miR-1204, miR-1205, miR-1206, miR-1207-5p, miR-1207-3p, and miR-1208. miR-1204 targets VDR to promote EMT and metastasis in breast cancer^47^. The molecular functions and effects of PVT1 in GC require further investigation. Further studies of these transcripts may provide novel insights into the functions of PVT1 in lymph node involvement.

Taken together, our findings reveal that PVT1 has a high expression level in the GC tissues of both Han and Uygur patients. The level of PVT1 in tissues can help to assess the risk of lymphatic metastasis in GC patients. It is also known to correlate with expression of c-myc. PVT1 can be a potential biomarker to predict the tendency for metastasis in both Han and Uygur GC patients.

In future research, because the exact regulatory mechanism for metastasis of PVT1 needs to be explored, we plan to analyze the PVT1-protein interaction networks in an attempt to identify the transcription factors or polymerases that are involved in the mechanism of PVT1 in GC cells.

## Materials and Methods

### Sample collection

We collected 42 GC tumor tissues from 22 Han and 20 Uygur patients, and 47 normal gastric tissues, from 23 Han and 24 Uygur. We also collected 95 serum samples from four different ethnic groups, including 54 Han, 21 Uygur, 14 Kazak and 6 Hui people. The samples were collected at the First Affiliated Hospital of Shihezi, Xinjiang, China and the First Peoples’ Hospital of Kashi, Xinjiang, China. Clinical data of GC patients and normal people were obtained by medical record review. The patient records and information were anonymized and de-identified prior to analysis. Details of the investigation and the required informed consent were reviewed and approved by the Ethics Committee of the First Affiliated Hospital of Medicine School, Shihezi University.

GC TNM staging was according to the AJCC/UICC^7th^ edition staging system^48,49^. T1 represents tumor invasion of the lamina propria, muscularis mucosae or submucosa; T2 represents tumor invasion of the muscularis propria; T3 representstumor penetration of subserosal connective tissue without invasion of visceral peritoneum or adjacent structures; T4 represents tumor invasion of the serosa (visceral peritoneum) or adjacent structures. GC *in situ* was categorized as stage 0; stage I includes T1N0M0 and T2N0M0; stage II includes T3N0M0, T2N1M0, T1N2M0, T4N0M0, T3N1M0, T2N2M0, and T1N3M0; stage III includes T4N1M0, T3N2M0, T2N3M0, T4N2M0, T3N3M0, and T4N3M0, and stage IV includes any T, any N and M1.

### Tissue Microarray (TMA) construction

All GC tissues were sectioned and stained with hematoxylin and eosin (HE). In addition, representative tissue areas of each sample were selected and marked on the slides. Subsequently, the fields corresponding to these selected regions were located in the corresponding paraffin block for TMA construction. Tissue cylinders of 1.0 mm diameter were punched from these areas of each donor tissue block and brought into a recipient paraffin block using a homemade semiautomated tissue arrayer (Alphelys, Plaisir, France). The region of each tissue represented at least 70% of the typical region of interest in that sample. Finally, 5 μm-thick serial sections were prepared from the TMA blocks for ISH and IHC.

### Detection of PVT1 expression in GC TMA by ISH

Paraffin-embedded tissue samples were cut into 4 μm-thick sections and mounted on slides. Samples were dewaxed in xylene and rehydrated using a graded series of ethanol solutions. Endogenous peroxidase activity was abolished by incubating the slides for 10 min in a peroxidase-blocking solution. After that, 0.1 M HCl was added to the GC TMA and incubated for 20 min, after which they were incubated with 0.5% Triton X-100 for 30 min. Sections were then treated with proteinase K solution, and finally fixed in 4% paraformaldehyde. Prehybridization was performed by adding 100 µL RNA hybridization solution dropwise. After pre-hybridization, 100 μL of probe was dropped onto the slide and it was hybridized overnight at 37 °C (18–20 h). The next day, anti-digoxin HRP-conjugated secondary antibody (dilution 1:100) was added and incubated for 2 h at 37 °C. The slides were then incubated in diaminobenzidine (DAB; Dako, Glostrup, Denmark)) buffer for 5 min, then stained with hematoxylin and finally washed with water. The slides were dehydrated through graded alcohol to xylene and mounted in an anti-fade mounting medium with mounting glass.

### Detection of c-myc expression in GC TMA by IHC

Paraffin-embedded tissue samples were cut into 4 μm-thick sections and mounted on slides. Tissue samples were dewaxed in xylene and rehydrated through a graded series of ethanol solutions. Endogenous peroxidase activity was abolished by incubating the slides for 5 min in a peroxidase-blocking solution. A total of 50 μL of the primary antibody against c-myc (ZSGB-BIO, Beijing, China, 1:100) was dropped onto each slide, followed by incubation overnight at 4 °C. The slides were then incubated with DAB (Dako) buffer for 5 min. Subsequently, slides were stained for 3 min with hematoxylin and then washed with water. The slides were dehydrated through graded alcohol to xylene and mounted in an anti-fade mounting medium with a mounting glass.

### ISH and IHC scoring criteria

All the immunostained slides were independently evaluated by two experienced pathologists. The expressions of these two markers were scored based on cytoplasmic/nuclear staining intensity and percentage of positively-stained cells. The staining intensity was categorized as follows: 0, negative; 1, buff; 2, yellow and 3, brown. The percentages of positively-stained cells were scored as follows: 0 (<5% positive cells), 1 (6–25% positive cells), 2 (26–50% positive cells), 3 (51–75% positive cells) or 4 (≥76% positive cells).

The percentages of positively-stained cells and the staining intensities were further multiplied to generate the ISH score for each case and to evaluate PVT1 and c-myc expression. Four categories of expression were listed as follows: − (a score of 0–1), + (a score of 2–4), ++ (a score of 5–8), and +++ (a score of 9–12). PVT1 and c-myc were considered negative when the score was between + and ++ categories, whereas the +++ scorecategory were considered PVT1 positive.

### Cell culture

The human GC cell lines AGS, SGC7901, N87, MGC803, BGC823 and MKN45 were donated by the Laboratory of Molecular Oncology^3^, Peking University Cancer Hospital/Institute. The cells were routinely cultured in DMEM containing 4.5 g/L glucose and supplemented with 10% fetal bovine serum (FBS). Cells were maintained at 37 °C in a humidified atmosphere of 5% CO_2_.

### RNA extraction and cDNA synthesis

Total RNA from GC cells and serum was isolated using Trizol reagent (Invitrogen, Carlsbad, CA, USA) following the manufacturer’s instructions. A 750 μL aliquot of Trizol reagent was added directly to 250 μL of serum. The integrity of RNA was confirmed using 1.2% agarose gel electrophoresis. The purity and concentration of RNA were assessed by measuring the absorbance by spectrophotometer at 280 and 260 nm. Total RNA from all samples was reverse transcription using a Revert aid First Strand cDNA Synthesis Kit (Thermo Fisher Scientific, Waltham, MA, USA) following the manufacturer’s protocol and every sample had an RNA content of 1.0 µg.

### Real-time PCR

The PVT1 expression level was quantified with a SYBR Green PCR kit (Qiagen, Valencia, CA, USA) following the manufacturer’s protocol, and the following primers: forward, 5′-GGAAGGTGGAGCGTAAGGA-3′ and reverse, 5′-CAATGCCGCCAATCTTGTA-3′. The length of the quantitative PCR product was 92 base pairs. The expression levels of PVT1 in each cell line were normalized to the respective β-actin expression levels with the following primers: forward, 5′-CCCAGCACAATGAAGATCAAGATCAT-3′, and reverse, 5′-ATCTGCTGGAAGGTGGACAGCGA-3′ (product length, 101 base pairs). The amplification protocol included an initial heat activation step at 95 °C for 5 min, followed by 40 cycles of denaturation at 95 °C for 30 s and a combined annealing/extension step at 55 °C for 30 s. The expression of PVT1 was calculated using the 2^(−ΔCT)^ value and the specificity of each reaction was confirmed by melting curve analysis.

### Plasmid transfection and RNAi assay

The shRNA interference plasmids for lncRNA PVT1 were synthesized by Sangon, Ltd (Shanghai, China) using the plasmid pGPU6/GFP/Neo. Four shRNA plasmids for PVT1 were synthesized and named sh-PVT1-1, sh-PVT1-2, sh-PVT1-3, and sh-PVT1-4 (Supplement Table 1), with the empty plasmid designated sh-NC. The interference sequence was 5′-GGACTTGAGAACTGTCCTTAC-3′. Human GC BGC823 and AGS cells were transfected with 2 µg sh-PVT1 or sh-NC using Lipofectamine 2000 transfection reagent (Life Technologies) according to the manufacturer’s instructions. After 48 h, cells transfected with shRNA were lysed and RNA was extracted for real-time PCR to determine the transfection efficiency (Supplement Fig. 2).

### Western blotting

Total proteins were collected from the GC cells. Proteins were fractionated by sodium dodecyl sulfate polyacrylamide gel electrophoresis, transferred to polyvinylidene difluoride membranes, blocked in 5% skimmed milk powder at room temperature for 2 h, and incubated with antibodies at 4 °C overnight. Antibodies against c-myc (mouse monoclonal antibody anti-c-myc; 1:100; Boster, Wuhan, China) and beta actin (mouse monoclonal antibody anti-β-Actin; 1:1000; ZSGB-BIO, Beijing, China). Next, the membranes were washed three times with TBST, and the secondary antibody (goat anti-Mouse IgG; 1:5,000; ZSGB-BIO) was then added and incubated for 2 h at room temperature, then washed three times with TBST.

### Cell Counting Kit-8 (CCK8) assay

To determine cell growth, 5 × 10^3^ cells were seeded into 96-well plates and transfected with shRNA. Cell proliferation was determined using CCK8 according to the manufacturer’s protocol. The fluorescence intensity was measured using a fluorescence microplate reader and absorbance was measured at 450 nm (Molecular Devices, Sunnyvale, CA, USA). Three independent experiments (three replicates in each) were performed.

### Invasion and migration assays

First, the interference plasmid of PVT1 was transfected into BGC823 and AGS cells for 48 h. The transfected cells were then treated with trypsin and counted, and 3 × 10^4^ cells were inoculated into a 24-well plate with 8 mm pore size chamber inserts. For invasion assays, Matrigel (1:8 dilution) was placed into the upper chamber and the plate was incubated for 3 h at 37 °C. Cells in 200 µL serum-free DMEM were seeded into a Transwell apparatus incubator at 37 °C in 5% CO_2_ for 48 h. DMEM containing 20% FBS (600 µL) was added to the lower chamber. For migration assays, cells were placed into the upper chamber of each well with the non-coated membrane and 20% FBS was added to the lower chamber; the plate was then incubated for 24 h. At the end of the incubation time, cells were fixed in methanol for 20 min, stained in 0.1% crystal violet for 30 min and subjected to microscopic inspection.

### Statistical analysis

The rank sum test was used to compare the differences between GC and normal serum cases. The correlations between PVT1, c-myc and clinicopathological characteristics of GC patients were analyzed by *χ*^2^ and Spearman’s test. All statistical analyses were performed using Statistical Products and Services Solutions software (SPSS, version 20.0, Chicago, IL, USA). Values of *P* < 0.05 were considered statistically significant.

## Supplementary information

Supplement table and Figures
