# Supplementary material for: High Expression of long non-coding RNA PVT1 predicts metastasis in Han and Uygur Patients with Gastric Cancer in Xinjiang, China
Source: Sci Rep. 2019 Jan 24;9:548. doi: 10.1038/s41598-018-36985-x (PMC6345741; doi:10.1038/s41598-018-36985-x)
Supplement: Supplementary file 1 — Supplement table and Figures [file 41598_2018_36985_MOESM1_ESM.pdf]

High Expression of long non-coding RNA PVT1 predicts metastasis in Han and Uygur Patients with Gastric Cancer in Xinjiang, China

Xianxian Ren[1+], Dongdong Cao[1+], Li Yang[1+], Xia Li[1], Lei Zhang[2],Yongbiao Xiao[3], Yu Xi[2], Feng Li[1,4], Dongmei Li[1*], Zemin Pan[1*]

[1]School of Medicine, Shihezi University, Shihezi, Xinjiang,China;

[2]First Affiliated Hospital of Shihezi University, Shihezi,Xinjiang, China;

[3]First people's Hospital of Kashi, Xinjiang, China;

[4]Dapartment of Pathology, Beijing Chaoyang Hospital, Capital Medical University, Beijing, China.

***Correspondence to:** Dr. Dongmei Li and Dr. Zemin Pan, Department of Biochemistry and Molecular Biology, School of Medicine, Shihezi University. North 2nd Road, Shihezi, Xinjiang, 832002, China.Tel: 86-993-2057882; Fax: 86-993-2057882; E-mail: lidong_abc@126.com; E-mail: panteacher89@sina.com.

[+] These authors have contributed equally to this work

Supplement table 1. The information of PVT1 plasmid interference sequence

Plasmids	Sequence
sh-PVT1-1	GCTTCTCCTGTTGCTGCTAGT
sh-PVT1-2	GCTTCAACCCATTACGATTTC
sh-PVT1-3	GGATTCTTACAGCTTGGATGT
sh-PVT1-4	GGACTTGAGAACTGTCCTTAC
sh-PVT1-NC	GTTCTCCGAACGTGTCACGT

Supplement Figure 1

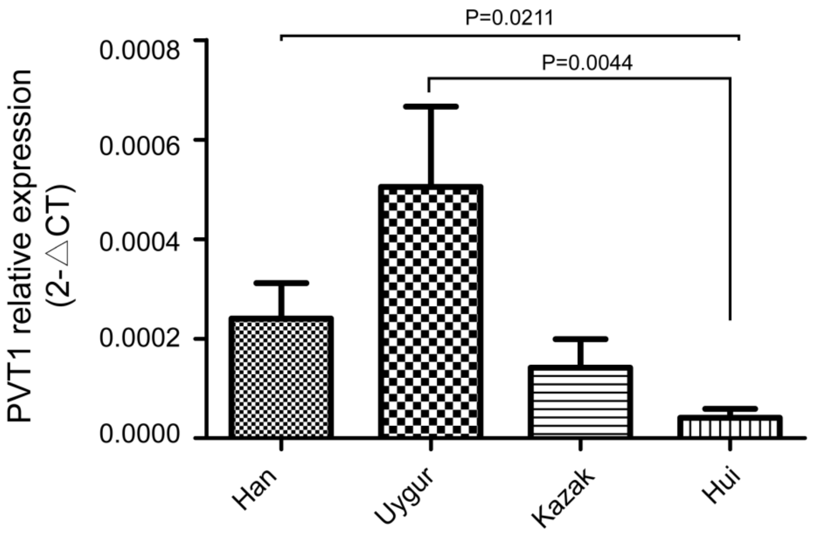

Supplement Figure 1. Detection of serum PVT1 level in four different ethnic groups.

Real-time PCR was used to detect the serum level of PVT1 in four different ethnic groups including Han, Uygur, Kazak and Hui, and to detect the basic expression levels of PVT1 in serum. PVT1 levels in Uygur people were higher than that in the other groups (*P*=0.0211). PVT1 serum levels in Uygur and Hui people were statistically significantly different (*P*=0.0044).

Supplement Figure 2

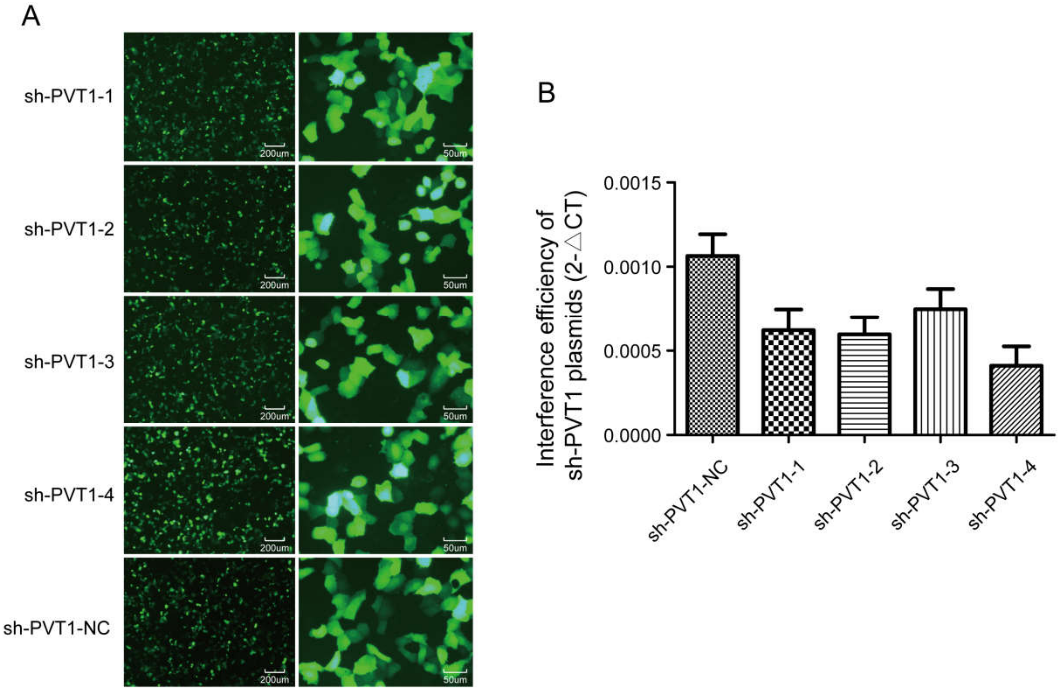

Supplement Figure 2. Detection of transfection and interference efficiency of different sh-PVT1 plasmids.

(A) Four sh-PVT1 plasmids, including sh-PVT1-1, sh-PVT1-2, sh-PVT1-3, sh-PVT1-4 and control plasmid sh-PVT1-NC were transfected in BGC823 cells. The GFP signal in cells at 40×and 400× magnification was detected to assess the success of transfection. (B) Real time-PCR was used to investigate the interference efficiency of different sh-PVT1 plasmids in BGC823 cells.
